# 

**Published:** 1883-12

**Authors:** 


### EDITORIAL.

### Close of the Volume.

This number closes the Thirty-seventh Volume of the DENTAL REGISTER. It is not now proposed to state that it is the oldest dental journal in existence, nor that it is the best dental journal in the world ; nor that it presents the best appearance ; nor that it is the only one published that is worth anything ; nor that it is the only representative journal ; nor that it is the only embodiment of all that is truly professional and scientific ; nor that it has a rapidly growing list of subscribers, and is already taken by every dentist on earth, and has a large list of subscribers in the Moon and in Jupiter ; nor that it has a subscription list of 2,005,006 ; nor is there an attempt to intimate that it is the only INDEPENDENT dental journal in the world, for that would not be true.

The Register is the farthest imaginable from INDEPENDENCE. It don't want to be independent.

It was dependent for its existence on its mother, the Mississippi Valley Dental Society. *It* had a mother.

It was dependent on its subscribers for the means of its support.

It also was dependent on advertisers (oh horrible) to help pay the printer, the paper maker, and the ——editor.

It is dependent upon the writers in the profession for contributions to its pages. It is dependent upon its friends and others for its circulation.

It is dependent upon societies, upon colleges, upon medical journals, and even upon dental journals.

Now, in the face of all this, it would be sheer nonsense, falsehood, and bunkum to claim that this is an independent journal.

Every now and again there is a howl for an independent dental journal, generally this comes from those who are suffering from professional atrophy.

The man who will establish a dental journal independent of everybody and anything shall have every possible encouragement by contributions—of wind.

The Register will be just as dependent for the coming year as it has been in the past, and in addition to the great labor heretofore bestowed upon it, is proposed to send a special agent to Madagascar and Wrangleland to secure 28,000 additional subscribers.

### Personal.

Dr. U. D. Billmeyer, D. D. S., of the Dental College of the University of Michigan, has resigned his position of Demonstrator of Clinical Dentistry in that institution, where he has served for several years with marked ability and success.

He leaves Ann Arbor for Chattanooga, Tenn., to engage in the practice of his profession there. The Dr. makes this change for a more congenial climate.

It is with sincere regret that those connected with the college part with Dr. B.; for, in addition to his being a fine operator, he is, in every respect, a gentleman.

The people of Chattanooga are to be congratulated, that Dr. B. has chosen to make their city his home.

May large success and prosperity attend him.

### Obituary.

### INDEX TO VOLUME XXXVII.

Addenda to the Report of a Case of Scurvy.................. 583

A Historical Poem.................. 591

A New Dental Disease............... 44

A New Dental College............ 47

Antiseptics........................... 83

A Case of Gangrene Oris.......... 89

A Centenarean........................ 96

Annual Commencement of the Dental Department of the University of Maryland..... 149

A Simple Stomatoscope............ 184

Address.................................. 222

American Medical Association.. 247

A New Disk.......................... 253

A Proposed Dental Reform...... 293

A Tooth at Birth..................... 287

A New Rubber Clamp.............. 304

A Suggestion—Journalistic...... 306

Anæsthetics—Use and Results... 338

Another Law Regulating Dental Practice .......................... 349

American Dental Association... 354

Alimentation among Savage and Civilized Peoples.............. 357

Address.................................. 395

American Dental Society of Europe ........................... 402

A Singular Case..................... 406

Absorbent Cotton.................... 408

Address of Dr. J. G. Fredericks 409

A Few Practical Observations on the Treatment of Pulp...... 412

American Dental Association... 416

A Synopsis of the Proceedings of the American Dental Ass'n. 433

Academy of Medicine in Ireland 448

A Substitute for Celluloid........ 451

Another Use for Carbolic Acid.. 457

Anæsthetics ........................... 467

A Case—Amalgam.................. 510

Amalgam as a Material for Filling Teeth ......................... 533

A Case of Plastic Surgery........ 543

Aesthetic Dentistry................. 548

Article on Dentition................ 553

A Peculiar Fracture of the Lower Jaw ................................ 557

Building Fine Houses on Sand... 385

Billroth's Clinic in 1882............ 452

Breakfast Beverages................ 556

Biography of Dr. T. B. Hamlin.. 296

Competitive Examination in China .............................. 96

Case of Deafness Probably Caused by Amalgam Fillings..... 133

Chloroform Water.................. 134

Central Illinois Dental Society... 144

Correspondence....................... 146

Change of Time...................... 198

Chicago Dental Election........... 202

Cerebral Despepsia.................. 246

Chemistry via. Dentistry........... 378

Close of the Volume..... ........ 609

Carbolized Iodoform............... 402

Case of Antral Abscess of Twenty Years' Standing................. 499

Clinics.................................. 528

Care of Teeth ........................ 562

Color Blindness.......................

Doctors of Medical Science...... 88

Decaigne on the Effects of Tobacco.......................... 91

Dental Department of the University of California......... 97

Disinfection by Hot Air............ 135

Degeneration of the Teeth........ 186

Dental Department of the University of Maryland........... 196

Dentinal Structure as Related to the Theory of Heitzmann............................ 122

### INDEX.

Dental Education .... 233

Description of a Novel Tooth Crown .... 331

Dressing Disks .... 353

Dental Pathology .... 383

Dental Law in Michigan .... 407

Danger of Spreading Disease by Books .... 455

Danger—Carelessness .... 459

Dental Nomenclature .... 451

Erosion of the Teeth Considered as a Retrospection Sign of Infantile Eclampsia .... 117

Extracts from Medical Clinic of Prof. Whittaker .... 129

Effects and Causes .... 203

Exposed Pulp and Treatment .... 219

Examinations .... 254

Etiology and Pathology of Dental Caries and Treatment of Pulp .... 255

Educational Association .... 304

Empiricism .... 315

Eyesight of School Children .... 344

Educational .... 354

Ear-ache in Children .... 401

Ether Spray in Facial Neuralgia 455

Effect of Alum Gargles upon the Teeth .... 502

Faith an Element of Success in Practice .... 85

Female Doctors .... 92

Female Brain-work .... 137

Germs and Acids in Decay of the Teeth .... 335

History of the Indiana Dental Association .... 99

Historical Sketch of the Mad-river Dental Society .... 189

Hymenial .... 303

Helps in Study .... 321

Half-breed Micro-organisms .... 493

Human Obesity .... 508

Health Aphorisms .... 560

How Shall We Blister? .... 561

Ho! for Niagara .... 356

In Memoriam .... 94

International Congress .... 145

Illinois State Dental Meeting .... 254

Indiana State Dental Meeting .... 301

In Memory .... 348

Inheritance as a Cause of Drunkenness .... 501

Insufficiency of Food in Certain Cases .... 503

Instrument Cases .... 504

Inodorous Iodoform .... 560

Kentucky State Dental Associa'n 303

" " " " 346

Loss of Both Parotid Glands .... 136

Leeches .... 291

Listre and the Spray .... 451

Mortality Statistics .... 50

Michigan State Dental Society .... 148

Meeting of Dentists at Louisville .... 194

Merited Honor .... 200

Mad River Dental Society .... 202

Missouri Dental Journal .... 254

Minutes of the Michigan Dental Association .... 273

Mixed Anæsthesia by Ether, Morphine, and Atrophine .... 289

Meeting—Mad River .... 305

Meeting of the Board of Examiners .... 349

Mechanical Dentistry .... 381

Malignant Pustule Communicated by a Fly .... 507

Medicine as Practiced by Animals .... 446

Metastic Chroditis After the Extraction of a Molar Tooth .... 555

N. O. D. Association .... 201

Note upon a Method of Root Filling .... 498

Nitrous Oxide and Chloroform as An Anæsthetic .... 505

Nomenclature .... 546

Notched Teeth .... 558

On Specialism; and on the Influence of Medical Science on Modern Civilization .... 1

On the Remedies Used to Promote Absorption of Inflammatory and other Morbid Products .... 9

Our Medical Literature .... 13

On the Relations which Dental Caries, etc .... 51

Ohio Dental College Association 99

On Certain Conditions of Dead Teeth .... 105

Office Light .... 138

Obituary .... 149

On Some Scientific Problems of Dental Surgery .... 151

INDEX.

Ohio College of Dental Surgery 198

On the Functions of the Nerves of Taste 261

On Nicotine and its Action on the Teeth 268

On the Conservative Treatment of Exposed Pulp 282

On Regulating Teeth 285

Obituary 305, 564

Operating for the Children 391

Ohio State Dental Society 511

Proceedings 139

Posteur's Response to Koch 249

Practical Applications of Koch's Discovery 250

Pepsin Preparations 404

Proceedings 561

Proceedings of the Ohio State Dental Society 596

Penn. State Dental Society 349

Questions Prepared and Adopted by the State Boards of Dental Examiners 428

Relative Mortality Amongst White and Colored Races 46

Researches on the Epithelium of the Cornea 93

Removal 201, 305

Retarded Growth 405

Remarkable Arrest of Development 457

Replantation of Teeth 533 565

Resorcin as a Dressing 560

Salasiylic Acid as a Prophylactic 44

Sanitary 49

Setting Questions 251

Something Practical about Micrococcie 450

Selections from a Clinical Lecture 453

Severe Hemorrhage After Tooth Extraction Treated by Transfusion 454

Sounds from the Consulting Room 458

Successful Exsection of Inferior Dental Nerve for Obstinate Neuralgia 491

State Board of Dental Examiners 512

Sandy Foundations 540

Sanitary 49

The Inervation of the Salivary Glands 43

Transmission of Traumatic Lesion 45

The Ohio State Dental College 48

The Medical and Dental Laws 49

The Physiology of Secretion 86

The Value of Antiseptics 87

Treatment of Scurvy 92

The Mississippi Valley Dental Association 98

Transactions 99

The Radical Cure of Alveolar Abscess 111

The late Dr. M. H. Webb 135

Thirty-ninth Annual Meeting of the Mississippi Valley Dental Society 162

There are Two 200

Transactions of the Ohio Dental Society 251

The Microscope 252

The Struggle for a Living 288

The late Dr. M. H. Webb 295

Transactions of the Association of State Boards of Dental Examiners 419

The Bi-Chromate Battery 456

Trichlorophenate of Lime 456

To Remove Fish Bones from the Throat 458

The Disinfecting Power of Antiseptics 487

Testing the Purity of the Atmosphere 489

The Effect of Tobacco on Youth 492

Treatment of Tumors by Electrolysis 502

The Poisons in Tobacco Smoke 504

The Effect of Alcohol on the Fœtus through The Blood of the Mother 506

The Medical Voyage of Life 508

The Conservative Treatment of the Dental Pulp, when Exposed, vs. Devitalization 513

University of Michigan 195

Unjustifiable Use of Chloroform 501

Writers' Cramp 12

Wheat Phosphates 30

Wants 100

Wm. H. Goddard 307

What should be done Further to Protect the Public from the Increasing Empiricism Practiced in our Large Cities? 326

Vertigo 401

### CONTENTS.

### COMMUNICATIONS.

Replantation .................. 565

Color Blindness .................. 574

Addenda to the Report of a Case of Scurvy .................. 583

A Historical Poem .................. 592

### PROCEEDINGS.

Proceedings of the Ohio State Dental Society .................. 596

### EDITORIAL.

Close of Volume .................. 609

Mutual Benefit Association .................. 610

Personal .................. 611

Obituary .................. 612

---

### THE DENTAL REGISTER,

A MONTHLY MAGAZINE DEVOTED TO THE INTERESTS OF THE

**DENTAL PROFESSION.**

*Terms Reduced to* $2.00 *Per Year. Single Copies,* 25 *Cents.*

**EDITED BY PROF. J. TAFT, D.D.S.**

AND

**Published by SPENCER & CROCKER, Ohio Dental and Surgical Depot.**

The volume commences in January and closes in December.

The REGISTER being issued monthly is always fresh, therefore Indispensable to the practitioner who desires to keep posted up with the new inventions and improvements which are daily developed. Neither expense nor effort will be spared to give value and variety to its contents Articles will be illustrated with well executed engravings whenever they will add to the interest or assist to explanation.

Its extensive circulation and frequency of issue make it one of the BEST DENTAL ADVERTISING MEDIUMS in the United States.

All subscriptions must begin with January or July.

Local or special notices, five lines or less, will be inserted for $3 each insertion; over five lines, 50 cts. per line.

All advertising bills payable in advance, or at furthest, after the issue of the first number containing the advertisement. All transient advertisements to be paid for in advance.

☞CONTRIBUTIONS SOLICITED.

Exclusively Editorial Communications should be addressed to the Editor.

The latest number of the REGISTER may be ordered with or without other goods at any time, and all letters should be addressed to

**SPENCER & CROCKER, Ohio Dental & Surgical Depot, Cincinnati, O.**

